# Study on Crystallization Behaviors and Properties of F-III Fibers during Hot Drawing in Supercritical Carbon Dioxide

**DOI:** 10.3390/polym11050856

**Published:** 2019-05-10

**Authors:** Xiaoma Ding, Haijuan Kong, Mengmeng Qiao, Zhifeng Hu, Muhuo Yu

**Affiliations:** 1 State Key Laboratory for Modification of Chemical Fibers and Polymer Materials, College of Materials Science and Engineering, Donghua University, Shanghai 201620, China; 1159124@mail.dhu.edu.cn (X.D.); 2160320@mail.dhu.edu.cn (M.Q.); 2160226@mail.dhu.edu.cn (Z.H.); 2 Shanghai Key Laboratory of Lightweight Composite, Shanghai 201620, China; 3 School of Materials Engineer, Shanghai University of Engineer Science, Shanghai 201620, China; Konghaijuan@sues.edu.cn

**Keywords:** F-III fibers, hot drawing, wide-angle X-ray scattering, small-angle X-ray scattering, mechanical properties, thermal stability

## Abstract

In order to obtain F-III fibers with high mechanical properties, pristine F-III fibers were hot drawn at the temperature of 250 °C, pressure of 14 MPa, tension of 6 g·d^−1^, and different times, which were 15 min, 30 min, 45 min, 60 min, 75 min, 90 min, and 105 min, respectively, in supercritical carbon dioxide (Sc-CO_2_) in this article. All the samples, including the pristine and treated F-III fibers, were characterized by a mechanical performance tester, wide-angle X-ray scattering (WAXS), small-angle X-ray scattering (SAXS), and thermogravimetric analysis (TGA). The results showed that the thermal stability of F-III fibers was enhanced to some extent, and the tensile strength and modulus of F-III fibers had great changes as the extension of treatment time during hot drawing in Sc-CO_2_, although the treatment temperature was lower than the glass transition temperature (Tg) of F-III fibers. Accordingly, the phase fraction, orientation factor f_c_ of the (110) crystal plane, fibril length l_f_, and misorientation angle B_φ_ of all the samples were also investigated. Fortunately, the hot drawing in Sc-CO_2_ was successfully applied to the preparation of F-III fibers with high mechanical properties.

## 1. Introduction

**Figure 1:**
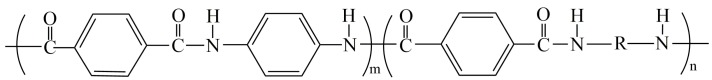
The molecular structure of F-III fiber.

Aramid fibers (AFs) have been widely used in bulletproof products, building materials, special protective clothing, electronic equipment, and other application fields owing to their super tensile strength, high elastic modulus, good impact resistance, great thermal stability, and excellent insulation property [1,2,3,4]. However, due to the rapid development of processing technology and harsh environment in which AF products are generally used, ordinary AFs need to be partially replaced by AFs with higher mechanical properties. F-III fiber, as one kind of AF, can be regarded as a block copolymer comprising three monomers (p-phenylenediamine, terephthaloyl chloride, and diamine containing a heterocyclic structure), which was developed by the Zhonglan Chenguang Chemical Research Institute. The molecular structure of the F-III fiber is shown in Figure 1. Compared with the current industrialized "para" AFs (such as Kelvar, Twaron, and Technora fibers) and "meta" AFs (such as Nomex and Conex fibers), the F-III fiber has shown higher tensile strength and modulus due to the more complex molecular structure. Due to the excellent mechanical properties, F-III fibers are specially used in the military bulletproofing field [5].

The supercritical carbon dioxide (Sc-CO_2_) fluid with the critical temperature and pressure of 31.1 °C and 73.8 bar (7.38 MPa) has confirmed to be a useful medium to induce crystallization during hot drawing in addition to the basic extraction and chemical reaction applications [6,7,8,9] due to it being easy to get, easy to remove, chemically inert, non-toxic, lack of pollution, strong diffusion capacity, and plasticization [10,11,12,13,14,15]. On the one hand, the Sc-CO_2_ can be dissolved into the polymers, increasing the flexibility of segments and providing plasticization, which reduces the glass transition temperature (Tg) of the polymers in the Sc-CO_2_ atmosphere [16]. On the other hand, the Sc-CO_2_ can induce the crystallization of fibers during hot drawing, thereby increasing the crystallinity of fibers. For example, Hobbs et al. used the Sc-CO_2_ as a reversible plasticizer, transport, and extraction medium to study the crystal annealing of commercial nylon-66, polyethylene terephthalate, and ultra-high molecular weight polyethylene fibers, respectively, during the post-treatment process, which showed that an increase in the modulus and toughness was achieved in nylon-66 [17]. Furthermore, Qiao et al. used the Sc-CO_2_ to induce the crystallization of polyacrylonitrile fibers, which showed that the crystallinity and mechanical properties of polyacrylonitrile fibers were improved after hot-drawing treatment [18].

Due to the many advantages of synchrotron radiation, its application in polymer research is more extensive [19]. Synchrotron radiation small-angle and wide-angle X-ray scattering (SAXS and WAXS) are important experimental methods for studying polymer crystals and other ordered structures [20,21,22]. Combined with the SAXS and WAXS methods, it is possible to simultaneously detect the structure of a kind of polymer with a size from 0.1 to 1000 nm. Many domestic and foreign experts have used SAXS and WAXS to study the internal crystallization behavior and ordered structure of AFs [23,24].

Hot-drawing treatment at a temperature above the Tg of AFs has accelerating effects on the crystallinity and degree of orientation of AFs [25,26,27]. The crystallization behavior can be employed as an important index to reflect the mechanical performance of AFs to some extent. Lots of researchers have conducted systematic experiments on the improvement of crystallinity and mechanical performance of AFs within specific treatment temperatures, tensions, and time ranges in the air and nitrogen atmospheres [25,26]. What’s more, it is obvious that the crystallinity of AFs increases with the extension of the treatment time [25].

The crystallinity and mechanical properties of AFs will not change too much during hot drawing in the air and nitrogen if the treatment temperature is below the Tg of AFs [28]. However, the tensile strength of AFs will be more or less damaged due to the high treatment temperature, although the treatment time is very short, resulting in the potential mechanical performance of AFs not being fully presented [29]. Therefore, we can try to prepare F-III fibers with high tensile strength and modulus through hot drawing by making use of the characteristics of the Sc-CO_2_ fluid at the temperature of 250 °C, which is below the Tg of F-III fibers. Additionally, our previous work has studied the effect of different pressures on F-III fibers, which showed that the mechanical properties of F-III fibers increased within the pressure range from 8 to 14 MPa, and then decreased at 14 MPa, so we chose 14 MPa as the experimental condition for the pressure in this experiment [5]. 

## 2. Materials and Methods

### 2.1. Materials

The pristine F-III fiber, which was composed of 150 monofilaments with the linear density of 44 tex and Tg of 275 °C, respectively, was supplied by the Zhonglan Chenguang Chemical Research Institute, Sichuan, China. Carbon dioxide (CO_2_) with a purity of 99.99% was purchased from Shanghai Junding Gas Co., Ltd., Shanghai, China. 

### 2.2. Hot-Drawing Process of F-III Fibers in Sc-CO_2_ Reactor

**Figure 2:**
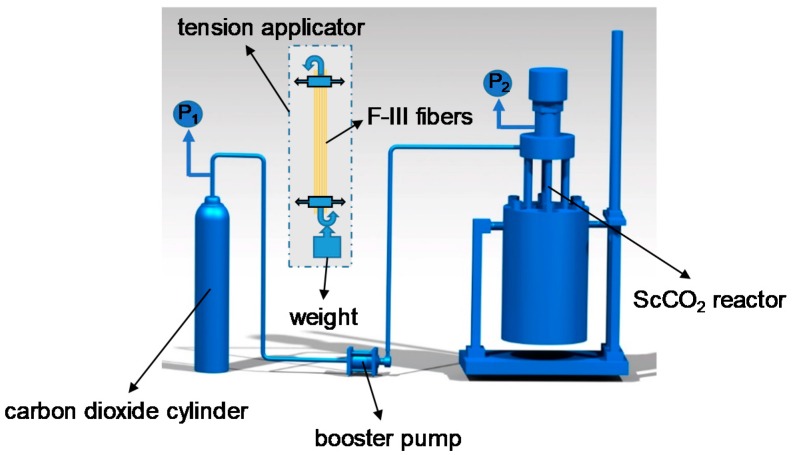
Schematic diagram of hot-drawing process of F-III fibers in the supercritical carbon dioxide (Sc-CO_2_) reactor.

Figure 2 shows the experimental equipment. The hot-drawing process is conducted in a 10-L Sc-CO_2_ reactor customized from Tianjin Yantu Experimental Instrument Development Co., Ltd., Tianjin, China. The hot-drawing process can be summarized as follows. Firstly, the Sc-CO_2_ reactor is heated to the temperature of 250 °C; after this, the pristine F-III fibers with a length of 200 mm under a tension of 6 g·d^−1^ (which was precisely controlled by the weights, as shown by the tension applicator part in Figure 2) are suspended in the Sc-CO_2_ reactor. Then, a small amount of CO_2_ gas is added to remove the air in the reactor. The CO_2_ gas output from the CO_2_ cylinder is transported to the reactor by a booster pump with a booster ratio of 60. Then, the CO_2_ gas is added into the reactor to 14 MPa, which was required for the experiment, and the temperature in the reactor was below 250 °C during the process of pressurization. Recording the reaction time when the temperature in the reactor reached 250 °C was required for the experiment again. Finally, the reaction lasted for 15 min, 30 min, 45 min, 60 min, 75 min, 90 min, and 105 min, respectively, at the temperature of 250 °C, pressure of 14 MPa, and tension of 6 g·d^−1^. The samples, including the pristine and treated F-III fibers, are collected for characterizations.

### 2.3. Characterizations 

#### 2.3.1. Mechanical Performance Test

The tensile strength, modulus, and elongation at break of samples are tested in a single-filament strength tester (XQ-1A, Shanghai New Fiber Instrument Co., Ltd., Shanghai, China) with a clamping distance of 20 mm and a stretching speed of 10 mm·min^−1^. The values of tensile strength, modulus, and elongation at break are the average of 30 valid test results.

#### 2.3.2. Wide Angle X-ray Scattering (WAXS) Measurement

A wide angle X-ray scattering (WAXS) measurement of each sample is implemented at the Shanghai Synchrotron Radiation Facility (SSRF) on a beam line (BL14B) with an X-ray wavelength dimension of 0.124 nm. The distance between the sample and detector (Mar 345) is 120.5 mm. The data analysis is performed by Xpolar software purchased from Precision Machinery Co., Ltd., NY, USA. There are crystal, mesomorphic, and amorphous phases in F-III fibers, and the fraction of different phases is obtained by peakfit software. We can calculate the crystallinity of F-III fibers according to Equation (1):

(1) $$\mathrm{CI}=\frac{A_{c}}{A_{c}+A_{m}+A_{a}}\times 100\%$$

where CI is the crystallinity of the F-III fibers, and A_c_, A_m_, and A_a_ are the fraction of the crystal, mesomorphic, and amorphous phases in the F-III fibers, respectively.

The orientation factor is an important indicator for evaluating the degree of orientation of fibers, which is calculated by the ordered crystals of certain crystal planes inside the fibers. In F-III fibers, the ordered structure of the (110) crystal plane is often used to reflect the degree of orientation. The orientation factor f_c_ of the crystal plane is calculated using Equation (2):

(2) $$f_{c}=\frac{3\cos^{2}\varphi -1}{2}$$

where φ is the angle between the fiber axis and the c-axis crystal unit. The orientation parameter (cos^2^φ) is confirmed on the basis of the Wilchinsky model. For example, for the reflection (110), the orientation parameter (cos^2^φ_110_) can be determined by Equation (3):

(3) $$\left(\cos^{2}\varphi \right)=\left(\cos^{2}\varphi_{110}\right)=\frac{\int_{0}^{\pi /2}I\left(\beta_{110}\right)\cos^{2}\beta_{110}{\sin\beta }_{110}{d\beta }_{110}}{\int_{0}^{\pi /2}I\left(\beta_{110}\right){\sin\beta }_{110}{d\beta }_{110}}$$

where β_110_ is the azimuthal angle of the (110) crystal plane, and I (β_110_) is the intensity of the azimuthal angle of the (110) reflection.

#### 2.3.3. Small Angle X-ray Scattering (SAXS) Measurement

A small angle X-ray scattering (SAXS) measurement of each sample was also implemented at the SSRF on a beam line (BL14B) with the same X-ray wavelength dimension as the WAXS. The difference from the WAXS is that the distance between the sample and detector (Mar CCD 165) is 1950 mm. The data analysis is performed by the uniform software. 

**Figure 3:**
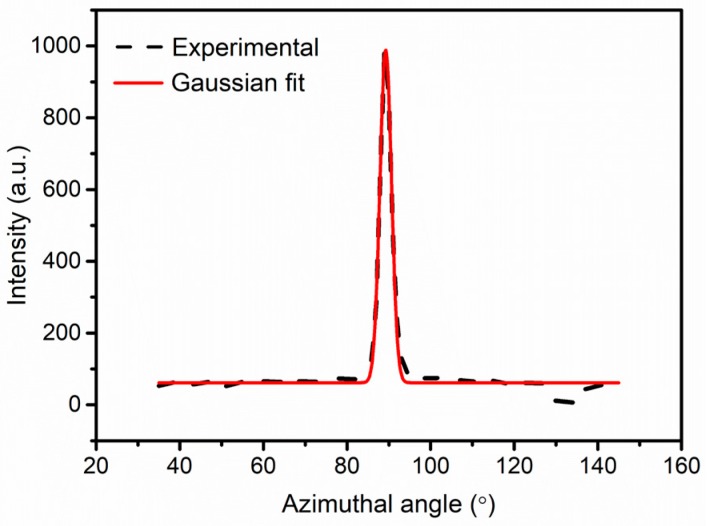
Azimuthal scan curve and corresponding Gaussian fitting curve.

**Figure 4:**
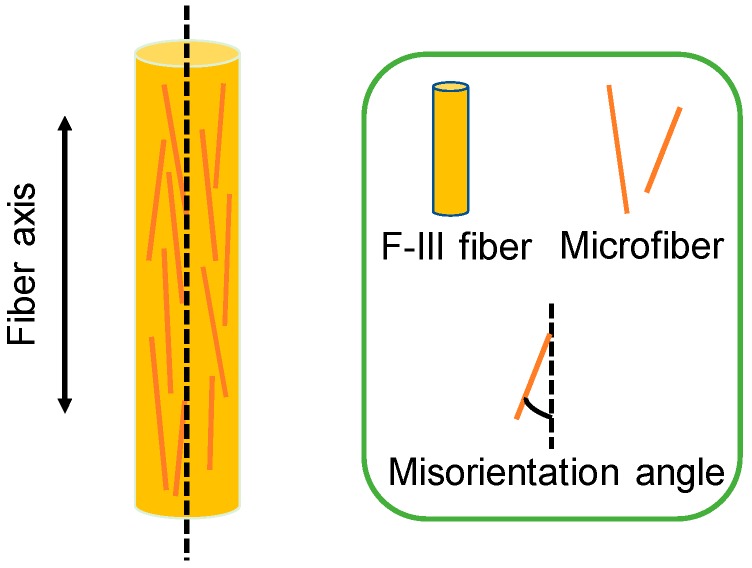
Schematic diagram of microfiber and misorientation angle in F-III fibers.

SAXS can be used to study the scattering phenomenon in the small angle range, and to analyze the changes in the internal structure of fibers within the micro-size, such as the length of the fibril or microvoids and the misorientation angle [24]. For F-III fibers, the interpretation of equatorial patterns in SAXS involves the microfiber with the absence of a lamellar structure and long period, which gives the orientation distribution of microfibers along the direction of the fiber axis [30,31]. Luo et al. pointed out that the scattering objects in F-III fibers were principally connected with the microfibrillar structure [30,31]. In addition, Ran et al. deduced that the scattering objects in Kevlar fibers were also relevant to the fibril structure [23]. Therefore, in this article, we are more inclined to believe that the microstructure of F-III fibers possesses a fibrillar structure by the way of the information from the literature and experiment analysis. For F-III fibers, the azimuthal scans of the equatorial streaks are according to the Gaussian function, as shown in Figure 3, which is applied to estimate the observed integral breadth B_obs_. In addition, for different scattering vector s, there is an association between the integral breadth B_obs_ and the scattering vector s. The angle between the microfiber and the fiber axis direction is defined as the misorientation angle B_φ_, as shown in Figure 4. The fibril length l_f_ and misorientation angle B_φ_ are calculated using Equation (4):

(4) $$s^{2}B_{\mathrm{obs}}^{2}=\frac{1}{l_{f}^{2}}+s^{2}B_{\varphi }^{2}$$

where s is the scattering vector, B_obs_ is the full width at the half-maximum of the azimuthal profile, and s can be determined by Equation (5):

(5) $$s=\frac{2\sin\theta }{\lambda }$$

where θ is the half value of the scattering angle 2θ, and λ is the wavelength dimension of the X-ray.

#### 2.3.4. Thermogravimetric Analysis (TGA)

The thermal stability of F-III fibers is studied by a thermogravimetric analysis (TGA, TG 209 F1 Netzsh, Selb, Germany) instrument. Both the shielding gas and purge gas are nitrogen, and the gas flow rates are 20 mL·min^−1^ and 30 mL·min^−1^, respectively. The heating rate is 10 °C·min^−1^, and the curves are recorded from room temperature to 900 °C.

## 3. Results and Discussion

### 3.1. Mechanical Performance Analysis

**Figure 5:**
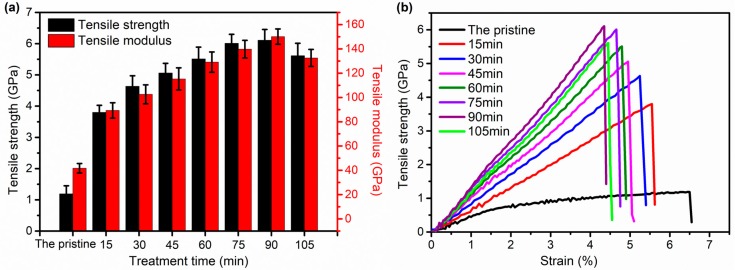
Tensile strength and modulus (**a**) and typical stress–strain curves (**b**) of the pristine and treated F-III fibers in Sc-CO_2_.

The tensile strength, modulus, and typical stress–strain curves of all the samples are shown in Figure 5a,b, respectively. The tensile strength and modulus of treated F-III fibers present an increasing trend when the treatment time is less than 90 min in Sc-CO_2_ fluid, and the tensile strength and modulus reach the maximum values of 6.1 GPa and 150.1 GPa, respectively, at 90 min. The main reason for this change is due to the improvement of the crystallinity and degree of orientation of F-III fibers as the extension of treatment time. However, the mechanical properties of F-III fibers begin to decrease when the time is longer than 90 min. Compared with F-III fibers treated at 90 min, the tensile strength and modulus of F-III fibers obtained at 105 min decrease by 8.2% and 11.7%, respectively. This is due to the interiors of F-III fibers being damaged to some extent when the treatment time is too long. The elongation at break, as an important indicator to measure the toughness of fibers, is depicted in Figure 5b, from which we can acquire that the elongation at break decreases before 90 min. In general, the greater the mechanical properties, the lower the elongation at break. This situation is similar to the fracture behaviors of other types of fibers reported in many literature studies [32,33,34,35,36,37].

### 3.2. Wide Angle X-ray Scattering (WAXS) Analysis

**Figure 6:**
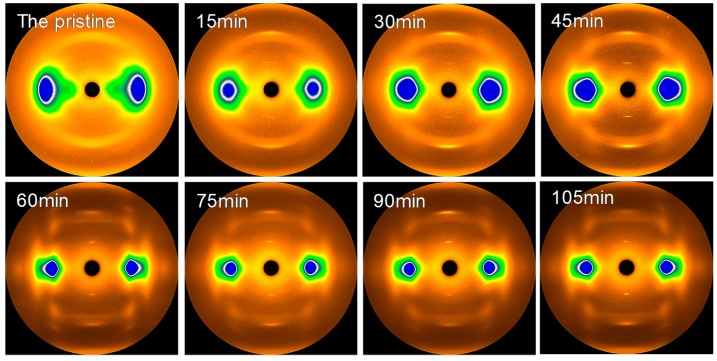
The two-dimensional (2D) wide angle X-ray scattering (WAXS) patterns of the pristine and treated F-III fibers in Sc-CO_2_.

The changes in the crystallization and ordered structure of F-III fibers during hot drawing in Sc-CO_2_ are analyzed by WAXS. The two-dimensional (2D) WAXS patterns of all the samples are depicted in Figure 6. The pattern of the pristine shows the gourd-shaped diffraction spots on the equator, implying a relatively ordered intermolecular filling in the transverse fiber axis; additionally, there are diffraction halos along the meridian, illustrating a poor sequence and orientation in the fiber axis. The three-dimensional (3D) crystalline structure comprising equatorial diffraction spots, meridional diffraction arcs, and some weak off-equatorial diffraction halos are acquired in F-III fibers after hot drawing. When the treatment time increases, the shape of the diffraction pattern obtained in the equatorial direction changes from a gourd shape with a blurred outline to a pentagon with a sharp outline. Similarly, the diffraction halos in the meridional direction change to clear diffraction arcs. At the same time, small diffraction spots appear on the inner ring. These changes all indicate an increase in the crystallization and ordered structure in F-III fibers. 

**Figure 7:**
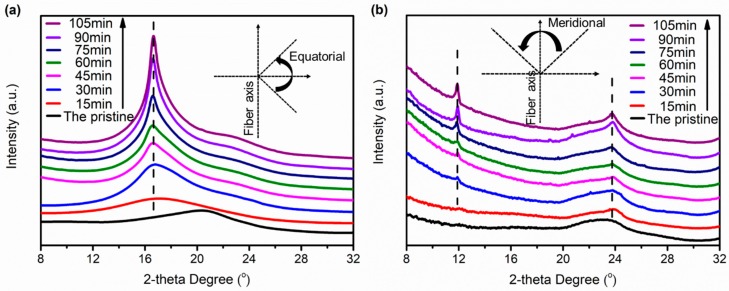
The integral diffraction intensities of wide angle X-ray scattering patterns of the pristine and treated F-III fibers in Sc-CO_2_: (**a**) equatorial direction, (**b**) meridional direction.

The radial integrations in the equatorial and meridianal directions of the 2D WAXS patterns of all the samples are shown in Figure 7a,b, respectively. The radial intensities of the equatorial and meridianal directions are acquired by integrating among −45° ≤ φ ≤ 45° and 45° ≤ φ ≤ 135°, respectively, where φ represents the azimuthal angle. The pristine possesses a wide diffraction peak at 2θ = 20.3° in the equatorial direction and 2θ = 23.4° in the meridional direction, respectively, indicating that the crystallinity of the pristine is relatively low. The position of the peak in the equatorial direction is shifted from 2θ = 20.3° to 2θ = 16.6° after hot drawing, indicating a larger d-spacing in treated F-III fibers. The intensity of the peak at 2θ = 16.6° corresponding to the (110) crystal plane tends to increase with the increase of treatment time before 90 min, implying the improvement of crystallinity in the equatorial direction. In addition, the position of the peak in the meridional direction is shifted from 2θ = 23.4° to 2θ = 23.8° after hot drawing, and the intensity of the peak at 2θ = 23.8° corresponding to the (004) crystal plane tends to increase with the increase of treatment time before 90 min. The treated F-III fibers show a new peak at 2θ = 12.0° which is corresponding to the (002) crystal plane, and the intensity of this peak increases with the increase of treatment time before 90 min. Both changes indicate the improvement of crystallinity in the fiber axis. This is due to the influence of the Sc-CO_2_ fluid and external tension: the Sc-CO_2_ fluid can reduce the force among chain segments, increase the flexibility of chain segments, and act as a plasticizer and solvent, so that the molecular chains can be easily rearranged in the direction of external force [38,39,40]. On the other hand, the Sc-CO_2_ and tension induce crystallizations in F-III fibers, and this effect is more obvious as the treatment time increases. However, when the treatment time exceeds 90 min, the intensity of the peaks in the equatorial and meridional directions begins to decrease, which is mainly because the internal crystal structure of F-III fibers will be destroyed owing to the excessive treatment time. On the whole, F-III fibers with high crystallinity can be acquired at a low temperature for a period of time in Sc-CO_2_ fluid.

**Figure 8:**
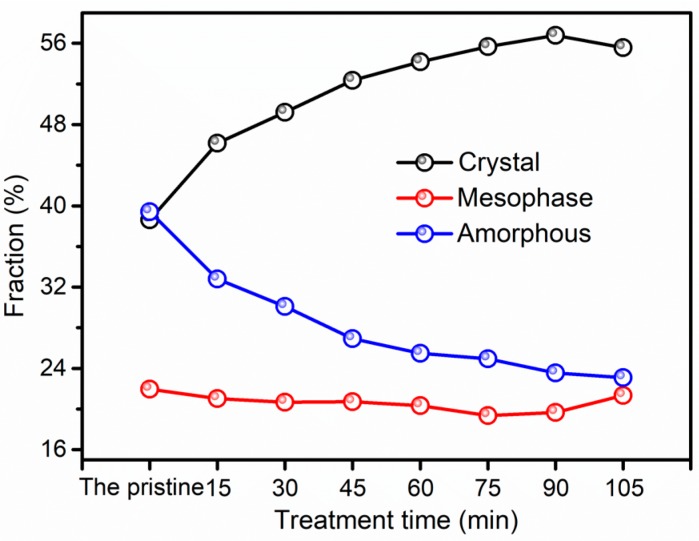
The crystal, mesomorphic, and amorphous fractions of the pristine and treated F-III fibers in Sc-CO_2_.

The crystal, mesomorphic, and amorphous fractions of all the samples are calculated by peakfit software, as depicted in Figure 8. The crystallinity of the pristine is 38.62%, and the crystallinity reaches the highest point of 56.77% at 90 min. Interestingly, the crystallinity begins to decline at 90 min, and the crystallinity of F-III fibers obtained at 105 min is decreased by 2% compared with F-III fibers acquired at 90 min. There is almost a ~19% to 22% fraction in F-III fibers considered as the mesophase, indicating that nearly ~19% to 22% of the molecular chains are in the intermediate phase, which is between the crystal and amorphous phases. According to the morphology and crystallinity reported in Kevlar fibers [41,42,43], we are not sure where the location of the mesophase is in the fibers. However, we can speculate that the mesophase is highly oriented molecular chains that are in the state of being able to crystallize, but there are lattice parameter defects. The amorphous phase fraction is on behalf of the boundary parts of the chains between the mesophase and crystal fibrils of the molecular chains, which represents the defect layer in fibers [23]. It exhibites a downward trend throughout the whole treatment time range. 

**Figure 9:**
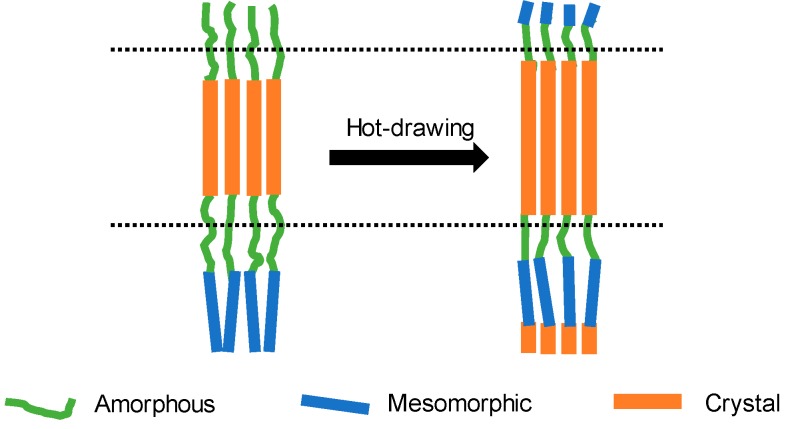
Schematic diagram of possible phase transitions in F-III fibers before and after hot-drawing treatment in Sc-CO_2_.

There is evidence that transitions among crystal, mesomorphic, and amorphous phases may occur. For example, it is found that a certain degree of change has taken place among the fractions of the crystal, mesomorphic, and amorphous phases during the hot-drawing process. The phase transition may occur during the hot-drawing process, as shown in Figure 9. Specifically, the amorphous region is transformed into the crystalline region and intermediate phase, and at the same time, the intermediate phase is also converted into the crystalline region. 

**Figure 10:**
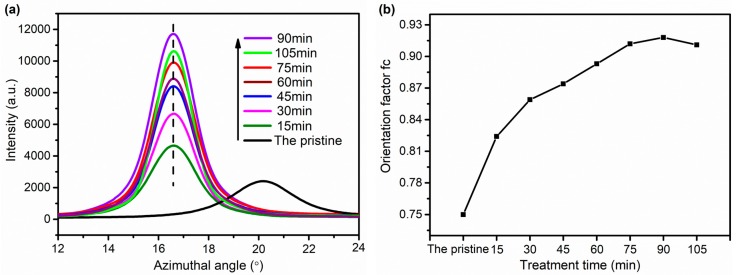
Azimuthal scan curves (**a**) and orientation factors (**b**) of the (110) crystal plane of F-III fibers before and after hot-drawing treatment in Sc-CO_2_.

The orientation factor f_c_ of the internal molecular chains in F-III fibers is calculated by scanning the full width at half maximum of the (110) crystal plane. The azimuthal scan curves of the (110) crystal plane and the orientation factor f_c_ of (110) crystal plane of F-III fibers are shown in Figure 10a,b, respectively. The orientation factor f_c_ increases with the increase of treatment time before 90 min, and then starts to decrease. This is because the inner molecular chains of F-III fibers undergo thermal motion, and the molecular chains preferentially move in the direction of the pulling force under the dual influence of the Sc-CO_2_ fluid and external tension, resulting in an increase in the orientation of the molecular chains along the axial direction of F-III fibers. However, when the treatment time is too long, the stable structure in F-III fibers is broken, and the microfibers in F-III fibers are broken to different degrees, resulting in a decrease in the degree of orientation of F-III fibers.

### 3.3. Small Angle X-ray Scattering (SAXS) Analysis

**Figure 11:**
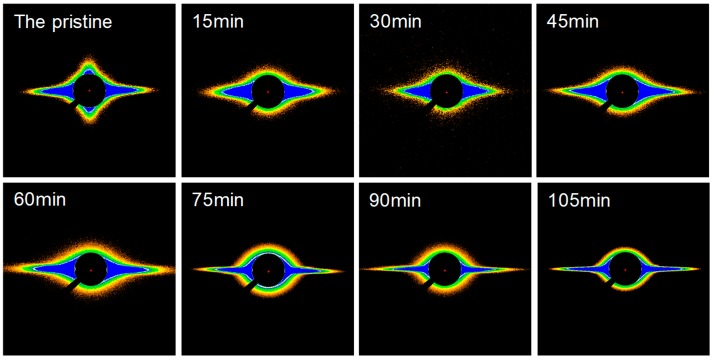
The small angle X-ray scattering patterns of the pristine and treated F-III fibers in Sc-CO_2_.

The microstructure of all the samples is analyzed at a large scale by SAXS. The SAXS patterns of the samples are shown in Figure 11. The pristine exhibites two symmetrical isosceles triangle streaks on the equator, and a little detectable scattering along the meridian. For the treated F-III fibers, the diffraction patterns in the meridian direction disappear, and at the same time, the apex angle of the symmetric isosceles triangle diffracted in the equatorial direction gradually decreases with the increase of treatment time less than 90 min. When the treatment time is longer than 90 min, the apex angle of the isosceles triangle begins to increase.

The fibril length l_f_ and misorientation angle B_φ_ are calculated according to Equation (4), as depicted in Table 1. Ran et al. reported that the fibril length l_f_ of Kevlar-49 was about ~77 to 90 nm [23], which was corresponding with our results. It is found that the fibril length l_f_ increases before 90 min, and then decreases, while the misorientation angle B_φ_ reduces continuously with the increasing treatment time less than 90 min, and then rises. For the changes of microfiber length l_f_ and misorientation angle B_φ_, it can be explained from the following aspects: firstly, as the treatment time increases, the molecular chains in F-III fibers are preferentially aligned along the fiber axis under the action of Sc-CO_2_ and tension, which may result in an increase in microfiber length l_f_ and a decrease in misorientation angle B_φ_. On the other hand, the transformation from the amorphous phase into the crystal may also lead to an increase in the length of the microfiber. However, as the treatment time further increases, the microfibers arranged in F-III fibers may be damaged by long-term hot drawing, resulting in a decrease in the length of the microfibers and an increase in misorientation angle B_φ_. However, the misorientation angle B_φ_ obtained at 105 min slightly increases compared with that at 90 min, indicating that the molecular chains of F-III fibers move slowly at a low temperature during hot drawing in Sc-CO_2_.

### 3.4. Thermogravimetric Analysis (TGA)

**Figure 12:**
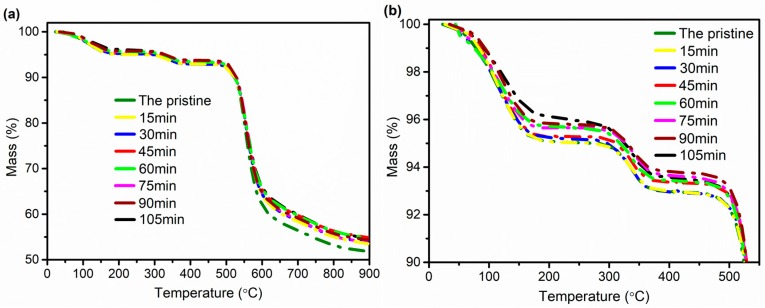
The thermogravimetric curves (~0–900°C) (**a**) and (~0–550°C) (**b**) of the pristine and treated F-III fibers in Sc-CO_2_.

The TGA method can be used to analyze the thermal stability of materials during heating and determine the working temperature of the materials. Figure 12 shows the thermogravimetric curves of F-III fibers before and after hot-drawing treatment. From the TGA curves, we can conclude that the F-III fibers have four thermal decomposition zones. The first interval is mainly the process in which F-III fibers lose the internal bound water. It can be seen from the curves that the quality of bound water in F-III fibers before and after hot-drawing treatment has no obvious changes. The second stage is the decomposition of small molecules in F-III fibers. The third interval is the thermal decomposition stage (~478–608 °C), and it is a severe degradation reaction, and the fourth interval is carbonization phase; at this interval, the F-III fibers are basically carbonized, and the heating process has little effect on the quality of F-III fibers [44,45]. 

In general, the initial decomposition temperature of fiber is defined as the temperature at which the mass loss of fiber is 5%. It can be concluded from Table 2 that as the treatment time increases, the initial decomposition temperature of F-III fibers gradually becomes higher, and the initial decomposition temperature of F-III fibers reaches 328.82 °C when the treatment time is 90 min. This indicates that the thermal stability of F-III fibers becomes better after hot drawing. The residual qualities of the treated F-III fibers are slightly larger than that of the pristine F-III fibers, but the small variation also indicates that the chemical structure of F-III fibers does not change after hot-drawing treatment.

## 4. Conclusions

In this experiment, F-III fibers with the maximum tensile strength and modulus of 6.1 GPa and 150.1 GPa, respectively, were acquired when the treatment time was 90 min during hot drawing in Sc-CO_2_. Compared with the pristine fibers, the crystallinity of F-III fibers increased by 47.0%, and the orientation factor of the (110) crystal plane increased by 22.4% at 90 min after hot drawing, which were obtained by WAXS. The SAXS showed that the fibril length of F-III fibers increased by 18.8%, and the misorientation angle decreased by 57.9% when the treatment time was 90 min compared with the pristine fibers. The TGA analysis showed that the initial decomposition temperature of F-III fibers increased from 281.32 °C to 328.82 °C bofore and after hot drawing at 90 min, while the changes of residual mass in F-III fibers were little, which indicated that no chemical changes happened in F-III fibers. These all indicated that hot drawing in Sc-CO_2_ was a useful method to prepare F-III fibers with high mechanical properties.

## Figures and Tables

**Table 1:** The fibril length l_f_ and misorientation angle B_φ_ of the pristine and treated F-III fibers in Sc-CO_2_.

Samples	Fibril Length l_f_ (nm)	Misorientation Angle B_φ_ (°)
The pristine	92.16	15.78
15 min	95.87	13.26
30 min	97.35	11.38
45 min	99.49	9.76
60 min	103.56	8.01
75 min	107.12	7.37
90 min	109.46	6.64
105 min	94.26	9.54

**Table 2:** The initial decomposition temperature and residual mass of the pristine and treated F-III fibers in Sc-CO_2_.

Samples	Temperature (°C, at Mass of 95%)	Residual Mass (%)
The pristine	281.32	51.82
15 min	282.84	53.52
30 min	296.49	53.69
45 min	307.31	54.86
60 min	316.36	54.72
75 min	324.36	54.35
90 min	328.82	54.16
105 min	326.86	54.44
